# Is there a best side for cochlear implants in post-lingual patients?^☆^

**DOI:** 10.1016/j.bjorl.2017.06.012

**Published:** 2017-07-29

**Authors:** Maria Stella Arantes do Amaral, Thiago A. Damico, Alina S. Gonçales, Ana C.M.B. Reis, Myriam de Lima Isaac, Eduardo T. Massuda, Miguel Angelo Hyppolito

**Affiliations:** a Universidade de São Paulo (USP), Faculdade de Medicina de Ribeirão Preto (FMRP), Departamento de Oftalmologia, Otorrinolaringologia Cirurgia de Cabeça e Pescoço, Ribeirão Preto, SP, Brazil; b Universidade de São Paulo (USP), Faculdade de Medicina de Ribeirão Preto (FMRP), Hospital das Clínicas, Setor de Fonoaudiologia, Ribeirão Preto, SP, Brazil; c Universidade de São Paulo (USP), Faculdade de Medicina de Ribeirão Preto (FMRP), Hospital das Clínicas, Departamento de Oftalmologia, Otorrinolaringologia e Cirurgia de Cabeça e Pescoço, Ribeirão Preto, SP, Brazil

**Keywords:** Cochlear implant, Hearing, Speech perception, Sound deprivation, Auditory residue, Implante coclear, Audição, Percepção da fala, Privação de som, Resíduo auditivo

## Abstract

**Introduction:**

Cochlear Implant is a sensory prosthesis capable of restoring hearing in patients with severe or profound bilateral sensorineural hearing loss.

**Objective:**

To evaluate if there is a better side to be implanted in post-lingual patients.

**Methods:**

Retrospective longitudinal study. Participants were 40 subjects, of both sex, mean age of 47 years, with post-lingual hearing loss, users of unilateral cochlear implant for more than 12 months and less than 24 months, with asymmetric auditor reserve between the ears (difference of 10 dBNA, In at least one of the frequencies with a response, between the ears), divided into two groups. Group A was composed of individuals with cochlear implant in the ear with better auditory reserve and Group B with auditory reserve lower in relation to the contralateral side.

**Results:**

There was no statistical difference for the tonal auditory threshold before and after cochlear implant. A better speech perception in pre-cochlear implant tests was present in B (20%), but the final results are similar in both groups.

**Conclusion:**

The cochlear implant in the ear with the worst auditory residue favors a bimodal hearing, which would allow the binaural summation, without compromising the improvement of the audiometric threshold and the speech perception.

## Resumo

**Introdução:**

O implante coclear é uma prótese sensorial capaz de restaurar a audição em pacientes com perda auditiva neurossensorial bilateral severa ou profunda.

**Objetivo:**

Avaliar se há um melhor lado para o implante coclear em pacientes pós-linguais.

**Método:**

Estudo longitudinal retrospectivo; incluiu 40 indivíduos, de ambos os sexos, idade média de 47 anos, com perda auditiva pós-lingual, usuários de implante coclear unilateral por mais de 12 meses e menos de 24 meses, com perda auditiva assimétrica entre as orelhas (diferença de 10 dBNA, em pelo menos uma das frequências), divididos em dois grupos. O Grupo A foi composto por indivíduos com implante coclear na orelha com melhor audição residual e Grupo B com menor audição residual em relação ao lado contralateral.

**Resultados:**

Não houve diferença estatística entre o limiar auditivo tonal antes e depois do implante coclear. Uma melhor percepção da fala nos testes pré-implante coclear foi observada no grupo B (20%), mas os resultados finais foram semelhantes em ambos os grupos.

**Conclusão:**

O implante coclear na orelha com pior audição residual favorece uma audição bimodal, o que possibilitaria a somação binaural, sem comprometer a melhora do limiar audiométrico e a percepção da fala.

## Introduction

A Cochlear Implant (CI) is a sensory prosthesis to restore hearing in bilateral severe-to-profound hearing loss if no Hearing Aid Devices (HAD) is effective. CI indications have been expanded to other types of losses due to technological advances related to software, devices and electrodes, and rehabilitation process.

For the indication of CI surgery, a multidisciplinary assessment is necessary, including audiological and imaging tests and etiological diagnosis. These tests are important to predict auditory responses after the speech processor is turned on.^1^

CI may be indicated for pre-lingual children with bilateral severe-to-profound sensorineural hearing loss or for post-lingual adults and children.^2^

Among the individuals with post-lingual deafness, some have audiometry with asymmetrical curves due to better hearing in one ear in comparison to the other and/or received higher asymmetrical hearing stimulus. After Computed Tomography (CT) image analysis and Magnetic Resonance Imaging (MRI) of the inner ear, excluding the indication of the best ear with good anatomical conditions, it is necessary to choose the side to the CI surgery in order to reach the best audiological results.^3^

Some studies indicate the CI device on the best hearing side with the best “hearing reserve” that represents more spiral ganglion cells surviving.^4^, ^5^, ^6^ Despite these considerations, patients who cannot have the bilateral implantation, choose CI surgery in the ear with worse hearing results with hearing aids.

Despite the increased possibility of surgical success on the side with better hearing reserve, it is known that unilateral CI provides monaural hearing, without the possibility of stimulation by a hearing device in the other side, limiting the location and sound discrimination in noisy environments.^7^

Aiming to offer the patient a possibility for binaural hearing, with bimodal adaptation, hearing aid stimulation in one ear and CI in the other one, was recommended by the International Consensus on cochlear implants in 2005 and some surgeons have chosen the side of poor hearing reserve for CI surgery in an attempt to provide hearing with binaural summation.^8^, ^9^, ^10^

Binaural hearing eliminates the shadow effect of the head, which is the obstruction of the head to the arrival of the sound stimulus when it is presented to one ear only; it provides the squelch effect, which is the ability of the auditory system to use the information from both ears when speech and noise are separated spatially and provides binaural summation as a result of central auditory processing to integrate and use the hearing of both ears.^11^

The aim of this study is to assess whether there is an indication for the best side for cochlear implants in post-lingual deafness patients.

## Methods

The study was approved by the Ethics Committee under number 56931916.8.0000.5440.

A retrospective longitudinal study was conducted using the review of medical records of post-lingual deafness patients undergoing cochlear implant surgery between 2004 and 2014 to evaluate the best audiological response variables such as age at the time of implant, gender, hearing loss time, stimulus time in each ear with hearing aids, sound deprivation time and audiological characteristics of each patient before and after CI were analyzed. To obtain the post-cochlear implant results, audiological results were standardized at the period from 1 to 2 years post-CI.

Unilateral CI users with at least one full year using the device, who presented severe to profound asymmetric post-lingual hearing loss and who were 18 years old or older were included in this study. We excluded bilateral CI users, patients with severe to profound hearing loss with pre-lingual hearing loss, younger than 18 years, neuro-psychomotor disorders or meningitis etiology with central nervous system impairment.

Patients were divided into two Groups (A and B) according to the implanted side, that is, Group A with patients implanted on the side with the best auditory reserve and Group B, with patients implanted on the side with the worst auditory reserve. Both groups were subdivided for analysis according to the time of hearing deprivation, with time of deprivation below or equal to 10 years and with time of hearing deprivation over 10 years. The auditory deprivation time criterion for dividing the groups was based on reports obtained in the literature.^12^, ^13^

Group A was composed of patients implanted at the best hearing reserve side, whose pure tone audiometry thresholds at 500 Hz were greater or equal than 10 dB and they reported better hearing at the implanted side. Group B was composed of patients with CI at the worst hearing reserve side, whose pure tone audiometry thresholds at 500 Hz were lower than 10 dB and they reported worse hearing at the implanted side.

Data were compared through the non-parametric Mann–Whitney statistical test, in independent samples (between groups) and through Wilcoxon Signed-Rank Sum Test, in paired samples (pre vs. post), as the data did not follow the normal distribution. Therefore, the JMP SAS software, version 10.0 (SAS Institute Inc., Cary, NC, USA) was used.

## Results

Group A was composed of 19 patients implanted in the best hearing side, average age of 49 years (48.6 ± 15.3), 9 men and 10 women. Group B was composed of 21 patients implanted in the worst hearing side, average age of 45 years (45.4 ± 15.1), 6 men and 15 women. There was no difference between the groups regarding gender (*p* = 0.2201, Chi square test) and age (*p* = 0.504, Student-*t* test).

The time of hearing loss did not differ between Groups A (20.1 ± 11.6 years) and B (22.1 ± 12.4 years) (*p* = 0.601). The same occurred with time of hearing deprivation, Group A (10.5 ± 10.2 years) and Group B (10.7 ± 14.0 years) (*p* = 0.589, Mann–Whitney test).

Table 1 shows the average audiometry threshold at 500 Hz in the pre- and post-CI groups. The variable “gain” is the difference between the two periods of time.

**Table 1:** Average audiometry thresholds (dB) at 500 Hz.

Group	Pre-CI	Post-CI	Gain	*p*-value
A – Best	104.2 ± 11.2	33.2 ± 8.0	71.1 ± 16.7	<0.0001^b^
B – Worst	106.4 ± 12.2	31.2 ± 10.0	75.2 ± 16.0	<0.0001^b^
*p*-values	0.468^a^	0.492^a^	0.505^a^	

a Comparison between groups, Mann–Whitney test.

b Comparison between pre- and post-CI – Wilcoxon Signed-Rank Sum Test.

There was a significant gain in hearing thresholds after CI for both Groups A and B, but when the groups were compared, there was no significant difference in pure tone audiometry at 500 Hz before and after CI or audiometric gain between the two groups (*p* = 0.468, *p* = 0.492 and *p* = 0.505).

By analyzing average hearing level (measured at 500, 1000, 2000, 3000 and 4000 Hz) before and after CI, there was no statistical difference between the Groups A and B (*p* = 0.321 and *p* = 0.635, Mann–Whitney test) and no difference in audiological gain between the two groups (*p* = 0.455). For each individual group, gain before and after CI surgery was significant (*p* < 0.0001 for Groups A and B – Wilcoxon test).

Regarding the speech perception tests pre-CI (% accuracy for trisyllable), a better percentage was observed in Group B before CI surgery, but no significant difference in both groups (*p* = 0.069, Mann–Whitney test). After one year of using the speech processor, there are no significant differences in both groups (*p* = 0.974). As for each individual group, statistical difference was observed in pre- and post-CI situation (*p* = 0.0005, Group A) and (*p* < 0.0001, Group B – Wilcoxon test).

At 500 Hz in Group A (best side), there was no difference between deprivation time below or equal to 10 years or over 10 years in the pre- and post-CI (*p* = 0.7525 and *p* = 0.2962), which was also observed in speech perception tests before and after CI (*p* = 0.0585 and *p* = 0.2051). We observed statistical difference in the speech perception gain comparing the time of sound deprivation (*p* = 0.0125) (Table 2).

**Table 2:** Audiological results of the best hearing reserve side (Group A), according to the hearing deprivation time (*p*-value Mann–Whitney test).

Hearing deprivation time of the best implanted side	Patients with hearing deprivation time shorter than or equal to 10 years	Patients with hearing deprivation time longer than 10 years	*p*-value (comparison between the two hearing deprivation times)
Average at 500 Hz pre-CI	106 dB	102.22 dB	0.7525
Average at 500 Hz post-CI	34.4 dB	31.66 dB	0.2962
Gain at 500 Hz	71.6 dB	70.56 dB	0.7543
Speech Perception Test – pre-CI – trisyllable words	0%	16%	0.0585
Speech Perception Test – post-CI – trisyllable words	91.11%	77.71%	0.2051
Speech Gain	91.11%	61.71%	0.0125

In spite of sound deprivation in Group B, no difference was found between patients with below or equal to 10 years and over 10 years at 500 Hz to pre- and post-CI (*p* = 0.1393 and *p* = 0.0784) and in the speech perception tests pre-CI (*p* = 0.2038) or speech perception gain (*p* = 1.0000) (Table 3). When speech perception tests and time of sound deprivation were compared, statistical difference was found (*p* = 0.0130) (Table 3).

**Table 3:** Audiological results of the worst hearing reserve side (Group B), according to the hearing deprivation time (*p*-value Mann–Whitney test).

Hearing deprivation time of the worst side for implant	Patients with hearing deprivation time less than or equal to 10 years	Patients with hearing deprivation time more than 10 years	*p*-value (comparison between the two hearing deprivation times)
Average at 500 Hz pre-CI	104.23 dB	110 dB	0.1393
Average at 500 Hz post-CI	28.07 dB	36.25 dB	0.0784
Gain at 500 Hz	76.16 dB	73.75 dB	0.7708
Speech Perception Test – pre-CI – trisyllable words	25%	9%	0.2038
Speech Perception Test – post-CI – trisyllable words	90%	70.85%	0.0130
Speech Gain	65%	61.85%	1.0000

The frequency average to 500, 1000, 2000, 3000 and 4000 Hz compared to the different time of hearing deprivation post-CI to Group B showed statistically significant difference (*p* = 0.0352) (Table 4), which did not occur to Group A (*p* = 0.4751) (Table 5).

**Table 4:** Audiological results of the worst residual hearing side, according to the comparison between mean frequencies and hearing deprivation time (Group B) (bold indicates *p*-value < 0.05 Mann–Whitney test).

Hearing deprivation time of the worst side implanted	Less than or equal to 10 years	More than 10 years	*p*-value (comparison between the two hearing deprivation times)
Average Auditory Level at frequencies 500–4000 Hz, pre-CI, in dB	111.38	111.38	0.1768
Average Auditory Level at frequencies 500–4000 Hz, post-CI, in dB	28.15	35.5	**0.0352**
Mean gain	82.23	75.88	0.5137

**Table 5:** Audiological results of the best residual hearing side, according to the comparison between Average of frequencies and hearing deprivation time (Group A) (*p*-value Mann–Whitney test).

Hearing deprivation time of the best side implanted	Less than or equal to 10 years	More than 10 years	*p*-value (comparison between the two hearing deprivation times)
Average Auditory Level at frequencies 500–4000 Hz, pre-CI, in dB	112.91	108.88	0.4738
Average Auditory Level at frequencies 500–4000 Hz, post-CI, in dB	32.73	30.63	0.4751
Mean gain	80.18	78.25	0.8237

## Discussion

The electronic device in CI is the most effective prosthesis in the medical history and is a unique sensory prosthesis to restore sensorial deprivation. CI indications have undergone evolutionary times and have depended on the technological development, on the improvement of surgical techniques and the training and qualification of interdisciplinary teams involved with all stages of the implantation process, which have allowed this technology to benefit people with varying degrees of hearing loss at different hearing deprivation times.^11^

By comparing Groups A and B in pre-CI, we observed no difference in the pre- and post-CI average values at 500 Hz; but we observed better performance in the perception test pre-CI (trisyllable) in Group B (Group A – 9% and Group B – 20%).

This finding may be explained by the fact that most individuals in Group A presented profound bilateral hearing loss, when frequencies from 500 to 8000 Hz were taken into consideration and these patients have been implanted in situation of worse bilateral reserve than in Group B. Despite the difference in the audiometric threshold, no statistical difference was found when the preoperative Speech Perception Test (SPT) results between the two groups were compared. Individuals from both groups performed well in the postoperative SPT (Group A – 85.25%; Group B – 83.5%), in line with the findings of Boisvert et al.^14^

This similar SPT results for both post-CI groups may be attributed to the fact that implanting the CI device in the patients’ worst hearing side – the worst hearing reserve side – allowed them to use the hearing aid device in the CI-contralateral side after the surgery, allowing bimodal hearing over at least one year after CI. This would allow a better perception or understanding of words that prove CI-unilaterally favoring central binaural stimulation of mechanisms that facilitate the best hearing performance for the worst ear, even if analyzed separately. Another aspect is that in our sample we assume that patients had the same hearing rehabilitation, which cannot be assured because this aspect cannot be completely controlled in a retrospective sample. The results presented in Table 2 show that there is an improvement in pure tone thresholds after surgery for both groups and that the comparison between the best side and the worst side implanted is no different as the pure tone thresholds in pre- or post-CI surgery. These findings are consistent with the results of Gantz et al.^15^ who, after 12 months of CI, claim there is no relationship between preoperative evaluation tests (audiological, electrophysiological and speech perception tests) and speech improvement regardless of the implanted ear side and deduce that the best understanding of words performed by the CI is due to central mechanisms that facilitate the hearing process for the worse ear and do not depend on the presence or absence of cochlear hair cells.

Unlike Firszt et al.,^9^ whose sample consisted of patients without the classic indication for CI given the residual hearing in the best ear, with the patients evaluated with conventional hearing aids adapted in the best ear, our indication criteria were related to severe to profound hearing loss, with deep loss in the worse ear. Lazard et al.^16^ performed the CI on the best or the worst side with no effect on post-CI performance confirming that the implant in the worst ear does not affect the post-cochlear implant results and the level of residual hearing in the best side has a positive influence on post-CI results when it is possible to use bimodal stimulation.

By comparing Groups A and B regarding time of sound deprivation, we observed an improvement in speech gain for the shorter sound deprivation time on Group A (*p* = 0.0125) and on Group B, we observed better performance on post-CI SPT in the group with shorter sound deprivation time (*p* = 0.0130), indicating that the greater the sound deprivation time, the worst negative correlation in postoperative SPT, as demonstrated by Portmann et al.^17^ Patients who have good memory for sounds and speech have better performance with the CI. Every 10% increase in accuracy in scores of statements corresponds to an increase of approximately 4.4% in the word recognition scores.^18^

We also observed a correlation between the years of sound deprivation when we implanted the side with worse hearing reserve to analyze the average hearing level post-CI, by averaging the tone audiometry at 500–4000 Hz (patients with greater privation time). They showed a worse sound in postoperative audiometric average.

These results show that the choice for the best hearing reserve or worse reserve does not interfere with audiological results presented in the post-operative when the patient is below 10 years of sound deprivation and shows what can actually interfere with post-CI result, even in post-lingual individuals, is hearing deprivation time over 10 years, and allows us to assume that the stimulation of the central auditory pathways is dependent only on the presence of sound stimulus and not its laterality.

Articles with level evidence 2a, 2b and 2c show that the benefit post-CI for speech perception seems to be more positively correlated to the integrity of the auditory central nervous system pathways than the functional status of the inner ear and auditory nerve. In patients with asymmetric hearing loss, when only one ear will be implanted, the best choice for the cochlear implant is the ear with worse hearing threshold.

It is worth highlighting the need for further studies in this area, with a larger number of subjects in order to ensure better understanding of the contribution of the contralateral ear to the CI in the obtained results.

## Conclusion

There was no statistical evidence when comparing Groups A and B in relation to the (better or worse) side with CI implant, in relation to the average values of the obtained auditory thresholds and the perception and speech test.

There was a significant difference between the studied groups when the time of hearing deprivation was compared.

The choice of implanting the internal IC device in the ear with worse auditory reserve and shorter time of sound deprivation may favor bimodal hearing, which would allow binaural summation without impairing the audiometric threshold improvement and speech perception.

## Conflicts of interest

The authors declare no conflicts of interest.

## References

[1] Bento R.F., Brito Neto R., Castilho A.M., Gómez V.G., Giorgi S.B., Guedes M.C. (2004). Auditory results with multichannel cochlear implant in patients submitted to cochlear implant surgery at Medical School, Hospital das Clínicas, University of Sao Paulo. Braz J Otorhinolaryngol.

[2] Bittencourt A.G., Ikari L.S., Della Torre A.A., Bento R.F., Tsuji R.K., Brito Neto R.V. (2012). Post-lingual deafness: benefits of cochlear implants vs. conventional hearing aids. Braz J Otorhinolaryngol.

[3] Tamplen M., Schwalje A., Lustig L., Alemi A.S., Miller M.E. (2016). Utility of preoperative computed tomography and magnetic resonance imaging in adult and pediatric cochlear implant candidates. Laryngoscope.

[4] Shiomi Y., Naito Y., Honjo I., Fujiki N., Kaneko K., Takahashi H. (1999). Cochlear implant in patients with residual hearing. Auris Nasus Larynx.

[5] Incesulu A., Nadol J.B. (1998). Correlation of acoustic threshold measures and spiral ganglion cell survival in severe to profound sensorineural hearing loss: implications for cochlear implantation. Ann Otol Rhinol Laryngol.

[6] Nadol J.B., Young Y.S., Glynn R.J. (1989). Survival of spiral ganglion cells in profound sensorineural hearing loss: implications for cochlear implantation. Ann Otol Rhinol Laryngol.

[7] Feuerstein J.F. (1992). Monaural versus binaural hearing: ease of listening, word recognition, and attentional effort. Ear Hear.

[8] Offeciers E., Morera C., Müller J., Huarte A., Shallop J., Cavallé L. (2005). International consensus on bilateral cochlear implants and bimodal stimulation. Acta Otolaryngol.

[9] Firszt J.B., Holden L.K., Reeder R.M., Cowdrey L., King S. (2012). Cochlear implantation in adults with asymmetric hearing loss. Ear Hear.

[10] Patki A., Tucci D.L. (2015). Choice of ear for cochlear implantation: implant the better or worse-hearing ear?. Laryngoscope.

[11] Hyppolito M.A., Bento R.F. (2012). Directions of the bilateral cochlear implant Brazil. Braz J Otorhinolaryngol.

[12] Geier L., Barker M., Fisher L., Opie J. (1999). The effect of long-term deafness on speech recognition in postlingually deafened adult CLARION cochlear implant users. Ann Otol Rhinol Laryngol Suppl.

[13] Nasralla H.R., Goffi Gomez M.V., Magalhaes A.T., Bento R.F. (2014). Important factors in the cognitive development of children with hearing impairment: case studies of candidates for cochlear implants. Int Arch Otorhinolaryngol.

[14] Boisvert I., McMahon C.M., Dowell R.C., Lyxell B. (2015). Long-term asymmetric hearing affects cochlear implantation outcomes differently in adults with pre- and postlingual hearing loss. PLOS ONE.

[15] Gantz B.J., Tyler R.S., Rubinstein J.T., Wolaver A., Lowder M., Abbas P. (2002). Binaural cochlear implants placed during the same operation. Otol Neurotol.

[16] Lazard D.S., Vincent C., Venail F., Van de Heyning P., Truy E., Sterkers O. (2012). Pre-, per- and postoperative factors affecting performance of postlinguistically deaf adults using cochlear implants: a new conceptual model over time. PLoS ONE.

[17] Portmann D., Felix F., Negrevergne M., Bourdin M., Lagourgue P., Coulomb-Faye F. (2007). Bilateral cochlear implantation in a patient with long-term deafness. Rev Laryngol Otol Rhinol (Bord).

[18] Gomaa N.A., Rubinstein J.T., Lowder M.W., Tyler R.S., Gantz B.J. (2003). Residual speech perception and cochlear implant performance in postlingually deafened adults. Ear Hear.

